# Directionally selective retinal ganglion cells suppress luminance responses during natural viewing

**DOI:** 10.1038/srep35708

**Published:** 2016-10-19

**Authors:** Maesoon Im, Shelley I. Fried

**Affiliations:** 1 Boston VA Healthcare System, 150 South Huntington Avenue, Boston, MA 02130, USA; 2 Department of Neurosurgery, Massachusetts General Hospital, Harvard Medical School, 50 Blossom Street, Boston, MA 02114, USA

## Abstract

The ON-OFF directionally selective cells of the retina respond preferentially to movement in a preferred direction, but under laboratory conditions they are also sensitive to changes in the luminance of the stationary stimulus. If the response of these neurons contains information about both direction and luminance downstream neurons are faced with the challenge of extracting the motion component, a computation that may be difficult under certain viewing conditions. Here, we show that during natural viewing the response to luminance is suppressed, leaving a relatively pure motion signal that gets transmitted to the brain.

Directionally selective (DS) ganglion cells of the retina report the direction of motion by spiking robustly to movement of an object in one direction (preferred) and sparsely to movement of the same object in the opposite (null) direction^1^,^2^,^3^. It is somewhat surprising therefore that such cells also spike robustly to changes in luminance without corresponding motion^1^,^2^,^3^,^4^,^5^ as responsiveness to two different visual features raises questions of how downstream circuits interpret the meaning of individual spikes, *e.g.* does a given spike convey information about motion or about luminance? The fact that other types of ganglion cell also respond strongly to stationary luminance changes^6^,^7^,^8^ raises the possibility that separation of the two neural signals could be accomplished by downstream circuits although the bandwidth limitations of the optic nerve would seem to argue against the transmission of duplicate information. Responsiveness to multiple features by DS cells has been largely ignored in the feature detector literature, curious given that the identification of directional selectivity helped shape the feature detector hypothesis^9^,^10^.

Responsiveness to multiple features by DS cells may be especially problematic during natural viewing. This is because the effectiveness with which DS cells extract motion is thought to be compromised by many different aspects of a complex visual scene. For example, if a portion of the moving object extends beyond the narrow boundaries of the dendritic field of a given DS cell, the responses to motion can be significantly suppressed^11^. Similarly, discontinuities in a single object or multiple objects moving simultaneously can also significantly reduce the response^12^,^13^. In addition, the spatiotemporal properties of the surround^14^, including the presence of motion^15^,^16^, can also diminish the response elicited by a moving object. Because all of these different factors can be present during natural viewing, the ability of DS cells to extract motion may actually be quite limited. Further, if the response to motion is in fact reduced during natural viewing the challenge of separating the motion signal from the luminance signal becomes even more difficult.

Here we explore the effectiveness of the DS circuitry in detecting both dynamic motion and static luminance changes without motion during natural viewing. We find that responses to changes in luminance are strongly suppressed in favor of a response that reliably reports direction of motion.

## Results

We studied responses of ON-OFF DS cells in the isolated rabbit retina (see Methods and Figs S1–S3) to natural movies (see Methods) which contained both moving objects and luminance changes (without corresponding motion). To isolate the responses to motion from the responses to luminance, the same movie was presented multiple times with its orientation rotated between consecutive presentations (0 to 330° in 30° increments): scenes from the movie that contained pure luminance changes should elicit highly symmetric responses across orientations while portions that contained motion would align better with the cell’s preferred direction during some rotations of the movie but not others and therefore should produce varying responses. Comparison of such responses to those that arise in response to conventional laboratory stimuli (i.e. moving bars) should provide insight as to the effectiveness with which motion is computed during natural viewing.

**Figure 1:**
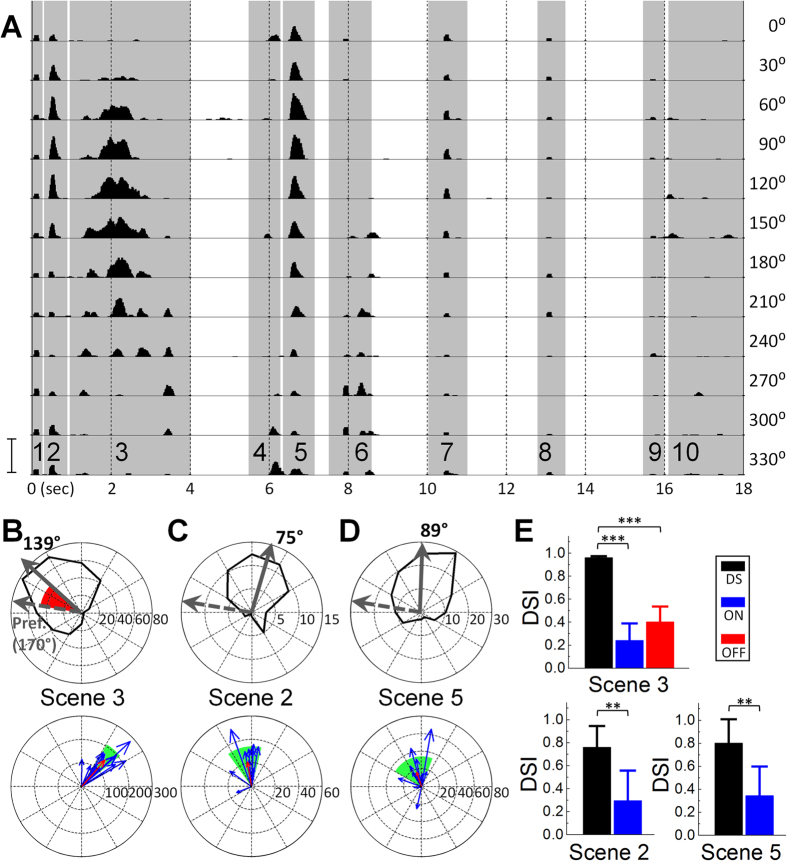
DS ganglion cells extract motion from natural scenes. (**A**) Peristimulus time histogram (PSTH) from a DS cell in response to a natural movie (Movie S1); each row corresponds to a different orientation of the movie (right). Shaded vertical bars correspond to individual scenes of the movie. Scale bar at bottom left (150 Hz) applies to all rows. (**B**–**D**) (Top) Polar plots of responses (spike counts) to Scenes 3, 2 and 5, respectively. Solid arrows are the vector sums of each plot; dashed arrows are the vector sums arising from moving bar responses. Arrow lengths not to scale. Red sector indicates the difference between the solid and dashed arrows and corresponds to the principal direction of motion for that scene (see text). (Bottom) Each blue arrow is the principal direction of motion detected by a single DS cell (*n* = 14); red arrow is the average of all cells. Shaded region in green indicates one standard deviation of the angular distribution. (**E**) Average direction selectivity indices (DSIs) computed from the population of DS (*n* = 14), ON (*n* = 7) and OFF (*n* = 8) cells. Unpaired one tailed student’s *t*-test was applied to verify the significance of statistical comparisons; **P < 0.01 and ***P < 0.001. Error bars, SD.

Consistent with previous work in non-DS retinal ganglion cells^17^ as well as in the lateral geniculate nucleus (LGN)^18^, responses to the natural movies generally consisted of sparse bursts of spikes (Fig. 1A) that occurred within distinct time periods. We refer to these distinct time periods as ‘Scenes’; individual Scenes in the first movie (Movie S1) ranged from 0.45–3.00 sec in duration (9–60 frames) and are indicated by shaded vertical bars in Fig. 1A and numbered 1–10 (bottom). Within several scenes, the strength of the response varied systematically with the orientation of the movie. For example, the level of spiking during Scene 3 was strongest for an orientation of 150° and got progressively weaker as orientation was stepped to 330°. When the number of spikes elicited during this Scene was plotted as a function of movie orientation (Fig. 1B, top), the asymmetry of the polar plot was comparable to that from polar plots generated by conventional moving bar stimuli (Fig. S1A, inset). The asymmetry in the polar plot from the natural movie did not arise from the properties of the movie itself as non-DS cells generated largely similar responses to different orientations of the movie (Fig. S4). Responses to several other Scenes with movement (*e.g.* 2 and 5) yielded similarly asymmetric plots (Fig. 1C,D, top). The direction selectivity indices (DSIs, see Methods and Fig. S2) for Scenes 3, 2 and 5 were 0.953 ± 0.030, 0.764 ± 0.184 and 0.804 ± 0.207 (mean ± SD, Fig. 1E*, n* = 14) and therefore comparable to the DSIs generated in response to moving bars (0.920 ± 0.071).

The strongest responses to movement within a natural scene should occur when the principal direction of the motion in the scene is aligned with the preferred direction of the cell. Thus for the cell in Fig. 1B, which had a preferred direction of 170°, the fact that the strongest response to Scene 3 occurred for an orientation of 139° suggests that the principal axis of motion in Scene 3 is 31° (the difference between the two, shown in red in Fig. 1B, top). Qualitative analysis of the video revealed this to be the case: an object that was centered at the start of the scene moves up and to the right at an angle of ~40° (Fig. S5). Because preferred directions can vary substantially from cell to cell, the optimum orientation of the movie is likely to vary for each cell but the angular difference between the optimum movie orientation and the cell’s preferred direction should remain approximately constant. This was indeed the case for each scene: angular differences were similar across cells and standard deviations were fairly narrow (Fig. 1B–D, bottom, the red arrows correspond to the averaged angular difference from all cells tested, *n* = 14). Taken together, these results suggest that DS cells reliably and accurately extract motion during natural viewing with an effectiveness that is comparable to that found with conventional moving bars.

**Figure 2:**
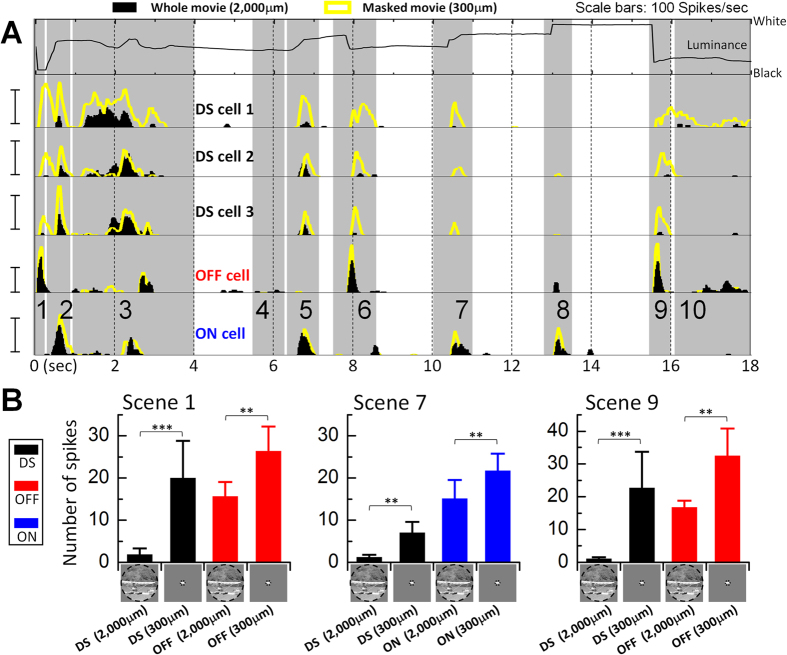
Luminance responses are suppressed during natural viewing. Responses to the full spatial extent of the movie (2000 μm diameter) were compared to responses when all but the central 300 μm of the movie was masked. (**A**) Row 1: the average luminance within the central 300 μm of the movie. Rows 2–4: PSTHs for preferred orientation responses in 3 typical DS cells; responses to the full movie (black) and the masked version (yellow) are overlaid. Rows 5 and 6: PSTH overlays for a typical OFF and a typical ON cell, respectively. All pairs of masked/unmasked responses were recorded within the same cell. Scale bars: 100 Hz. (**B**) Average numbers of spikes elicited during Scenes 1, 7 and 9 for full and masked movies. In DS cells (*n* = 8), responses were reduced by 92, 87 and 95% (Scenes 1, 7 and 9, respectively) (masked vs. unmasked). Responses of OFF cells (*n* = 6) were reduced by 41 and 49% for Scenes 1 and 9 while ON cells (*n* = 8) were reduced by 32% for Scene 7. Responses of a single cell were averaged across 12 orientations of the movie. Paired one tailed student’s *t*-test was applied to verify the significance of statistical comparisons; **P < 0.01 and ***P < 0.001. Error bars, SD. The movie frame shown at the bottom of bar charts is from ‘*CORAL REEF ADVENTURE*’ © MacGillivray Freeman Films. Used by permission. All rights reserved. This figure is not covered by the CC BY license.

It is well established that DS cells also respond strongly to sudden increases or decreases in luminance within their receptive field centers, *e.g.* to the onset and offset of stationary spots of light^1^,^2^,^3^,^4^,^5^, suggesting that sudden stationary luminance changes within a natural scene should produce a strong response as well. To explore whether this was indeed the case, we calculated the average luminance within the central 300 μm circular region of the movie as a function of time (top row of Fig. 2A). This region corresponds to the approximate size of the DS cell’s dendritic field^7^ and therefore allowed us to examine the relationship between luminance changes within the central portion of the cell’s receptive field and the resulting spiking responses. Surprisingly, the responses arising from many of the sudden changes in luminance were quite weak. For example, the sudden luminance decrease that occurred during Scene 9 was quite substantial yet generated little or no spiking (Fig. 2A, rows 2–4, black histograms). A similar paucity of spiking was noted for the luminance changes that occurred during Scenes 1 and 7. The lack of spiking in DS cells was not simply the result of an ineffective stimulus because these same scenes elicited robust spiking in both OFF and ON non-DS cells (Fig. 2A, black histograms in rows 5–6, respectively). These results suggest that responses to luminance changes are suppressed in DS cells during natural viewing.

To explore this suppressive effect further, an equi-luminant annulus was used to mask all but the central 300 μm of the movie (Fig. S6). With this mask in place, DS cells now generated strong spiking responses to the luminance changes of Scenes 1, 7 and 9 (Fig. 2A, rows 2–4, compare yellow traces to black histograms, Movie S1). Across the population of DS cells tested (*n* = 8) the reduction of spiking (1-unmasked/masked) was 92, 87 and 95% for the three scenes, respectively (Fig. 2B). Once again, these differences did not arise from a specific feature of the movie as similar results were obtained with a second natural movie (Movie S2); responses to a luminance change were reduced by 84% (*n* = 6) when the full movie was compared to the masked surround (compare responses at *t* = 12 sec, shaded vertical bars in Fig. S7A,S7B). Thus, these results suggest that the suppression of luminance responses in DS cells is mediated within the cell’s receptive field surround. Responses to luminance changes within the natural movie (S1) were also suppressed in non-DS cells (unmasked vs. masked) but the reduction was smaller leaving responses that were still quite substantial (Fig. 2B).

**Figure 3:**
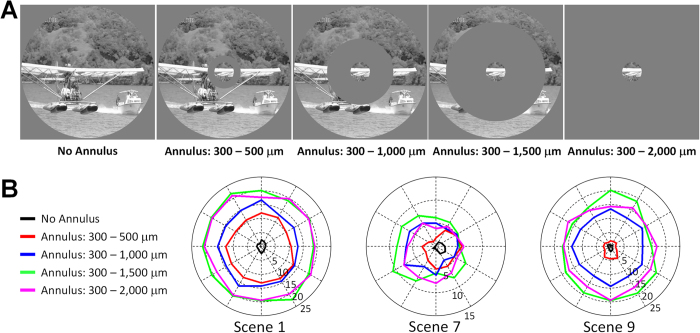
Luminance responses are suppressed by surround. (**A**) Annuli were used to mask increasing portions of the surround. The inner diameter of all annuli remained fixed at 300 μm while the outer diameter ranged from 500 to 2,000 μm. The movie frame is from ‘*CORAL REEF ADVENTURE*’ © MacGillivray Freeman Films. Used by permission. All rights reserved. This figure is not covered by the CC BY license. (**B**) Average responses (*n* = 5) to the luminance changes in Scenes 1, 7 and 9 for each mask size.

We explored the spatial extent of the region contributing to the observed suppression of luminance responses in DS cells by varying the size of the annulus used to mask the surround (Fig. 3A); the inner diameter of the mask remained fixed at 300 μm while the outer diameter was varied from 500 μm to 2000 μm (full mask). For the luminance changes associated with Scenes 1, 7 and 9 of the first natural movie (S1), the strength of the response increased steadily as increasing portions of the surround were masked (Fig. 3B, *n* = 5; see Fig. 2B and associated text for amount of suppression observed in each scene). Surprisingly, an increase in the response was observed even when the outer diameter of the mask was increased from 1000 to 1500 μm, suggesting that the annular region between radii of 500 and 750 μm from the soma of the DS cell also contributes to luminance suppression. As Fig. 3B suggests, the level of suppression was independent of the direction of motion.

To eliminate the possibility that specific features of the natural (unconstrained) movies were somehow biasing the suppression of luminance, we replaced the natural movie with a well-controlled laboratory stimulus consisting of a 300 μm spot of light, sized to optimally fill the center of the receptive field, and coupled it with a drifting grating that was simultaneously projected onto the receptive field surround; the annular shape of the grating had an outer diameter of 2000 μm and an inner diameter that ranged from 300 μm to 2000 μm (Fig. S8A). Consistent with the results derived from natural movies, DS cell responses to the stationary spot flash much larger when presented in isolation vs. when the drifting grating in the surround was included (Fig. S8B, S8C, filled bars; left-most filled bars show reductions of 83 and 64% for ON (*n* = 12) and OFF responses (*n* = 10), respectively), confirming that the surround of the DS cells strongly suppresses the response to stationary luminance changes. When the same experiment was repeated in non-DS cells, luminance responses were reduced but the magnitude of the induced suppression was significantly smaller than that observed in DS cells (compare open- and filled-bars in Fig. S8B, S8C, *n* = 10 and 8 for ON and OFF cells, respectively). Thus, there is an effective suppression of responses to luminance changes in DS cells that is mediated from the cell’s receptive field surround, and further, the suppression is distinct from the more conventional surround mechanisms known to exist in non-DS cells.

**Figure 4:**
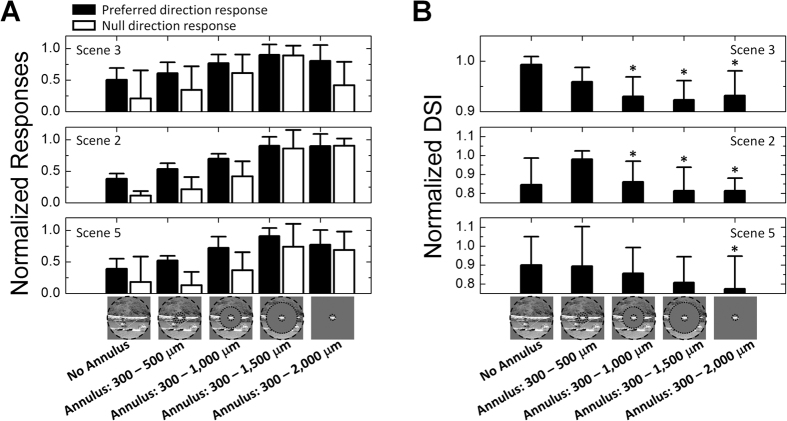
Natural viewing maximizes index of directionality. (**A**) Average responses (*n* = 5) to motion Scenes (3, 2 and 5) as a function of annulus size. Annuli shown in Fig. 3A were used to mask increasing portions of the surround. Filled and unfilled bars indicate responses to preferred and null direction responses, respectively. Each individual response was normalized by the maximum for that scene across all 12 orientations for that cell. Preferred and null responses were normalized independently of each other. (**B**) Average directional selectivity index (DSI) as a function of annulus size (*n* = 5). Within each plot, all DSIs were normalized to the maximum value for that scene. Error bars, SD. Paired one tailed student’s *t*-test was applied to verify the significance of statistical comparisons; *P < 0.05. T-test was performed between each column with the column showing biggest average of DSIs. The movie frame shown at the bottom of bar charts is from ‘*CORAL REEF ADVENTURE*’ © MacGillivray Freeman Films. Used by permission. All rights reserved. This figure is not covered by the CC BY license.

In addition to suppressing luminance responses, the surround of natural scenes also helped to optimize motion responses in DS cells. Similar to the approach with luminance responses, we compared responses to masked vs. unmasked natural movies for scenes that had strong motion components. Analogous to the luminance results, responses to both preferred and null direction motion generally increased as larger portions of the surround were masked (Fig. 4A, filled and unfilled bars, respectively; *n* = 5)^3^,^11^,^12^,^13^,^14^,^15^,^16^. However, the DSI arising from each of the different size annuli was found to decrease with increasing mask size (Fig. 4B, *n* = 5), resulting in DSIs that were highest for the full (unmasked) movie (Fig. 4B). Thus, even though the full extent of the movie reduced the overall magnitude of the response to motion, it enhanced the difference between preferred and null responses thereby resulting in the higher index. This implies that during natural viewing the DS system trades off a lower level of total spike output in an individual DS cell in exchange for a higher signal-to-noise ratio.

## Discussion

Our study provides three important new insights about the computations performed by DS cells. First, the use of natural movies revealed a suppression of the response to those luminance changes that occurred without associated motion (Figs 2 and 3). Further testing with well-controlled laboratory stimuli revealed that this suppression was mediated within the receptive-field surround (Fig. S8). The effectiveness of suppression was approximately uniform regardless of the direction of motion in the surround (Fig. 3B). The identification of luminance suppression helps to resolve a long-standing question about how downstream neurons distinguish between spikes intended to convey motion information and spikes intended to convey luminance information. By suppressing the response arising from stationary luminance changes, the surrounding circuitry ensures that DS cells transmit information about moving stimuli only. Second, we show that natural viewing leads to an enhancement of the directional index (Fig. 4B). This occurs because a wide-field inhibitory signal suppresses the response to movement. This suppression reduces the preferred-direction response slightly but reduces the null-direction response to almost nil (Fig. 4A). Coupled with the suppression of the response to luminance, this reduction of motion responses results in spike generation for only a single feature: motion of an object in the preferred direction. Responsiveness to only a single feature allows for unequivocal interpretation of transmitted spikes by downstream circuits and makes DS cells a true ‘feature detector.’ This finding is in contrast to the results of a recent study in which direction coding was not strongly affected by wide-field inhibition^19^. Although the changes to the DS index in our study were modest, they arose consistently when the surround inhibition was blocked and thus highlight the importance of natural scenes for extracting certain characteristics from visual neurons^20^,^21^,^22^,^23^,^24^,^25^,^26^,^27^. Third, we show that the computation of directional selectivity remains efficient during the viewing of complex natural scenes (Fig. 1). While this seems somewhat intuitive, much previous work suggested that many of the elements found in natural scenes would reduce the effectiveness of the directional computation^11^,^12^,^13^,^14^,^15^,^16^. Instead, we found the efficacy with which motion direction was extracted during natural viewing was comparable to that found with high-contrast moving bars or other conventional laboratory stimuli.

Because the spatial and temporal properties of natural stimuli differ markedly from those of artificial stimuli such as spots and bars^28^,^29^,^30^,^31^ the use of artificial stimuli may not be optimal for identifying all of the response properties of visual neurons, which presumably evolved to extract distinct visual features in the natural world. Accordingly, previous studies have used natural stimuli to identify new characteristics of visual neurons; this work has been done at all stages of the visual system. For example, natural stimuli were repeatedly found to be more effective in driving visual neurons^21^,^22^, mapping receptive fields^23^, and modeling neuronal responses^24^,^25^. The present study provides an additional example in which previously-unreported characteristics of visual neurons were identified from the use of natural stimuli. Our study thus supports the continued use of natural scenes to investigate the circuitry and mechanisms of direction selectivity and may help to unravel long-standing questions in the field such as those that pertain to the computation of direction or those that strive to better identify the functional role of these cells.

Our results do not reveal the neuronal source(s) that underlie the suppression of luminance but several inferences can be surmised from the above experiments. First, because suppression could be observed for distances up to 750 μm from the soma of the DS cell it is unlikely that this effect is mediated by starburst amacrine cells (SACs)^4^,^32^. If we estimate the dendritic field size of DS cells and SACs as 300^7^ and 400 μm^33^ respectively, the extent over which SACs could deliver a suppressive signal to DS cells is limited to ~350 μm from the DS soma^4^,^32^ (Fig. S9) – well below the actual extent for which suppression was observed. In addition, the lack of sensitivity of the suppressive mechanism to the direction of motion (Fig. 3B) further argues against a role for SACs^4^,^32^. Thus it is likely one or more wide-field amacrine cells^34^ play a role in the generation of suppression.

Our results do not reveal the source of the observed surround suppression to DS cells but comparison to previous studies offers some inferences. There is evidence for different surround mechanisms in DS vs. non-DS cells. Hoggarth *et al*.^19^ showed that GABAergic wide-field amacrine cells (WACs) mediate surround inhibition in the DS circuitry^19^ making these cells a potential candidate for the suppression observed here. Further support arises from the fact that the surround inhibitory signal was sensitive to tetrodotoxin (TTX) in DS cells^19^ but not in other types of ganglion cells^35^,^36^,^37^,^38^ and thus could underlie the different levels of luminance response suppression observed here (Fig. 2B). Poly-axonal amacrine cells (PACs) have axonal processes that extend >1 mm^16^,^39^,^40^; coupled with the fact that they are thought to supply an inhibitory signal to the DS circuit^16^ makes them another potential candidate for the surround inhibition observed here. Other possibilities exist as well including vesicular glutamate transporter 3 (vGluT3) amacrine cells (ACs) which have been shown to regulate the responses of DS cells^41^,^42^ and to receive strong inhibition from a wide surround^41^. The long-range effects of gap junctions between some subtypes of amacrine cells^40^ and nearby DS cells^43^ could also contribute. It will be interesting in future testing to elucidate the identity of the specific neurons involved in luminance suppression as well as the synaptic pathways that contribute to this effect.

## Materials and Methods

### Animal preparation and retina isolation

The care and use of animals followed all federal and institutional guidelines, and all the experiment protocols were approved by the Institutional Animal Care and Use Committees of the Massachusetts General Hospital and the Boston VA Healthcare System. New Zealand White Rabbits (~2.5 kg) were anesthetized with intramuscular injections of xylazine/ketamine and subsequently euthanized with an intracardial injection of sodium pentobarbital. The eyes were enucleated immediately after death. All procedures following eye removal were performed under dim red illumination in order to preserve photoreceptor function. After hemisecting the globe, the front of the eye was removed and the vitreous was eliminated. The retina was separated from the choroid and two rectangular pieces from the region just ventral to the visual streak (~5 mm) were prepared. The retina tissue was mounted, photoreceptor side down, to a 10-mm square piece of Millipore filter paper (0.45 μm HA Membrane Filter) that was mounted with vacuum grease to the recording chamber (~1.0 ml volume). A circle of ~2.1 mm in diameter at the center of the Millipore paper allowed light from below to be projected onto the photoreceptors.

### Electrophysiology

Patch pipettes were used to make small holes in the inner limiting membrane, and retinal ganglion cells (RGCs) were targeted under visual control. Spiking was recorded with a patch electrode (4–8 MΩ) that was filled with superfusate and positioned onto the surface of a targeted ganglion cell (cell-attached mode). Data were recorded and low-pass filtered at 2 kHz using Axopatch 200B amplifier (Molecular Devices, Sunnyvale, CA), and digitized at 10 kHz by NI-DAQ (PCI-MIO-16E-4, National Instruments). Two silver-chloride-coated silver wires served as the ground and were positioned at opposite edges of the recording chamber, each approximately 15 mm away from the targeted cell. The retina was continuously perfused at 4 ml/min with Ames solution (pH 7.4) at 36 °C, equilibrated with 95% O_2_ and 5% CO_2_.

### Cell type classification

RGCs were classified as ON or OFF cells by their responses to 1-sec-long stationary flashes (white spots on gray background) that ranged in size from 100 to 1,000 μm in diameter. They were classified as DS or non-DS by their responses to bright bars (300 μm × 1,800 μm at 600 μm/sec, white bars on gray background) that moved in 12 directions. The long axis of the bar was oriented parallel to the direction of movement. Light stimuli were generated by a digital light projector (LP120, InFocus, Portland, OR) and were focused onto the photoreceptors. The light stimuli and data acquisition were controlled by custom software written in LabView (National Instruments) and MATLAB (The MathWorks); Daniel Freeman developed an earlier version of the software.

All DS cells that elicited spikes in response to both the leading and trailing edges of a moving bar also responded to both the onset and offset of a small stationary flashed stimulus, and are therefore more precisely referred to as ON-OFF DS cells^2^. Another class of previously-reported DS cells, referred to as ON DS^2^, responds only to the onset of a flashed stimulus and was excluded from this study. In the present work, the abbreviation DS refers exclusively to ON-OFF DS cells.

Prior to presentation of movies, targeted ganglion cells were first classified as DS if their responses to a series of moving bars (Fig. S1) yielded a direction selectivity index (DSI, see Data analysis and Fig. S2) of 0.7 or larger for both the leading (ON) and trailing (OFF) edges of the bar (population responses shown in Fig. S3).

### Natural movie presentation

The naturalistic movie referred to in the text (Movie S1) is the official movie trailer of ‘*CORAL REEF ADVENTURE*’ (MacGillivray Freeman Films, Laguna Beach, CA) and was obtained from the internet. Another natural movie used in Supplementary Information (Movie S2) is part of an animal documentary of ‘World’s Deadliest: Stoat Hypnotizes Rabbit’ and was obtained from the webpage of National Geographic Society. These movies were converted to 8 bit (256 steps) gray scale in order to eliminate any potential bias that might arise from color sensitivity. The average luminance as a function of time for the central 300 μm of the movie, corresponding to the estimated size of the receptive field of DS cells used here^7^, was computed by averaging pixel brightness within this region for each frame. Natural images are known to have the linear profile in log-log plot of power spectrum^28^,^29^,^30^,^31^. With images captured from the natural movie we presented (Movie S1), the characteristic profile was obtained in a plot of the power spectrum (Fig. S10). In contrast, artificial images exhibit power spectrum profiles (Fig. S11) that were readily distinguishable from those of natural images.

The movies were presented 12 times (20 frames/sec, 18 sec for Movie S1 and 16 sec for Movie S2) to each cell; between each presentation the movie was rotated by 30 degrees. The natural movie (540 pixels in diameter) covered a circular area of 2 mm in diameter on the dissected retina (1 pixel = 3.7 μm). In rabbits, 2 mm in the retina is equivalent to approximately 11.8° in visual space (0.17 mm/°)^44^.

### Drifting grating test

A subset of RGCs was tested with complex laboratory stimuli (Fig. S8), consisting of a stationary spot flash, 300 μm in diameter, and presented for 1 sec in the receptive field center while a drifting grating was simultaneously presented over the surround. The drifting grating consisted of a square wave grating (2 cycles/mm) that was moving continuously at 2 cycles/sec. Spike counts were averaged separately across responses that were rotated in 12 directions. Annuli of various sizes were used to mask portions of the grating (Fig. S8A); luminance of each annulus was set to 128 (0 and 255 for black and white, respectively).

### Data analysis

Collected data were analyzed off-line using custom routines written in MATLAB (The MathWorks, Natick, MA). Peristimulus time histograms (PSTHs) were made with a bin size of 100 msec and a rolling step size of 20 msec. Polar plots were drawn for moving bar responses and movie responses. The preferred direction was calculated as the vector sum of the number of spikes for all twelve orientations; the number of spikes was averaged across three to five trials. The preferred directions for ON and OFF responses were calculated separately and then averaged to generate the preferred direction for the cell. All DS cells used in this study had differences in the ON vs. OFF preferred direction of <~20°. Two additional DS cells were omitted from further study because the difference between the preferred direction angles was >~30°.

To quantitatively assess the motion detection performance of recorded RGCs, we analyzed the directionality of responses with a direction selectivity index (DSI) which was calculated as:


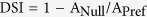


where A_Null_ and A_Pref_ are the area of the null-side half and the preferred-side half in the polar plot (Fig. S2). All fourteen ON-OFF DS cells used in this study had DSIs > 0.7 for both ON and OFF responses (Fig. S3) in response to moving bars; the fourteen cells were taken from thirteen different retinas. All non-DS cells had DSIs < 0.3. Non-DS cells included seven ON cells (obtained from seven retinas) and eight OFF cells (obtained from six retinas). In general, we targeted only those non-DS cells that exhibited brisk spiking responses to a flashed spot of light. In preliminary testing, we found little difference between non-DS cells with more transient light responses and non-DS cells with more sustained responses and so we did not attempt to further classify targeted cells.

Spikes elicited by the natural movies were generally sparse^17^,^18^ and therefore individual bursts could be easily separated into ten distinct scenes, each separated by a silent interval (Movie S1). For the analysis, spike counts were integrated over the following time intervals: Scene 1, 0.00–0.45 sec; Scene 2, 0.45–1.00 sec; Scene 3, 1.00–4.00 sec; Scene 4, 5.50–6.40 sec; Scene 5, 6.40–7.00 sec; Scene 6, 7.50–8.70 sec; Scene 7, 10.00–11.00 sec; Scene 8, 12.80–13.50 sec; Scene 9, 15.50–16.00 sec; Scene 10, 16.00–18.00 sec. We generally tried to avoid non-DS cells that had very sustained light responses as their responses to certain Scenes from the natural movie could also be prolonged thus making it difficult sometimes to unequivocally assign some spikes to a specific Scene from the natural movie.

### Statistical analysis

Unless mentioned otherwise, all data are given as the mean ± standard deviations (SD). Unpaired (Fig. 1E) and paired (Figs 2B, 4B, S7C, S8B and S8C) one tailed student’s t-tests were applied to verify the significance of statistical comparisons. Statistical significance is denoted in figures by * for *P* < 0.05, ** for *P* < 0.01 and *** for *P* < 0.001.

## Additional Information

**How to cite this article**: Im, M. and Fried, S. I. Directionally selective retinal ganglion cells suppress luminance responses during natural viewing. *Sci. Rep.* **6**, 35708; doi: 10.1038/srep35708 (2016).

## Supplementary Material

Supplementary Information

Supplementary Movie S1

Supplementary Movie S2
