# Supplementary material for: Directionally selective retinal ganglion cells suppress luminance responses during natural viewing
Source: Sci Rep. 2016 Oct 19;6:35708. doi: 10.1038/srep35708 (PMC5069630; doi:10.1038/srep35708)
Supplement: Supplementary Information [file srep35708-s1.pdf]

Supplementary Information for

"Directionally selective retinal ganglion cells suppress luminance responses during natural viewing"

Maesoon Im[1,2,†] and Shelley I. Fried[1,2]

[1]Boston VA Healthcare System, 150 South Huntington Avenue, Boston, MA 02130, USA

[2]Department of Neurosurgery, Massachusetts General Hospital, Harvard Medical School, 50 Blossom Street, Boston, MA 02114, USA

†Present address: Department of Ophthalmology, Henry Ford Health System, 1 Ford Place, Detroit, MI 48202, USA; Department of Anatomy and Cell Biology, Wayne State University School of Medicine, 540 East Canfield Street, Detroit, MI 48201

Correspondence and requests for materials should be addressed to M.I. (email: mim1@hfhs.org) or S.I.F (email: fried.shelly@mgh.harvard.edu).

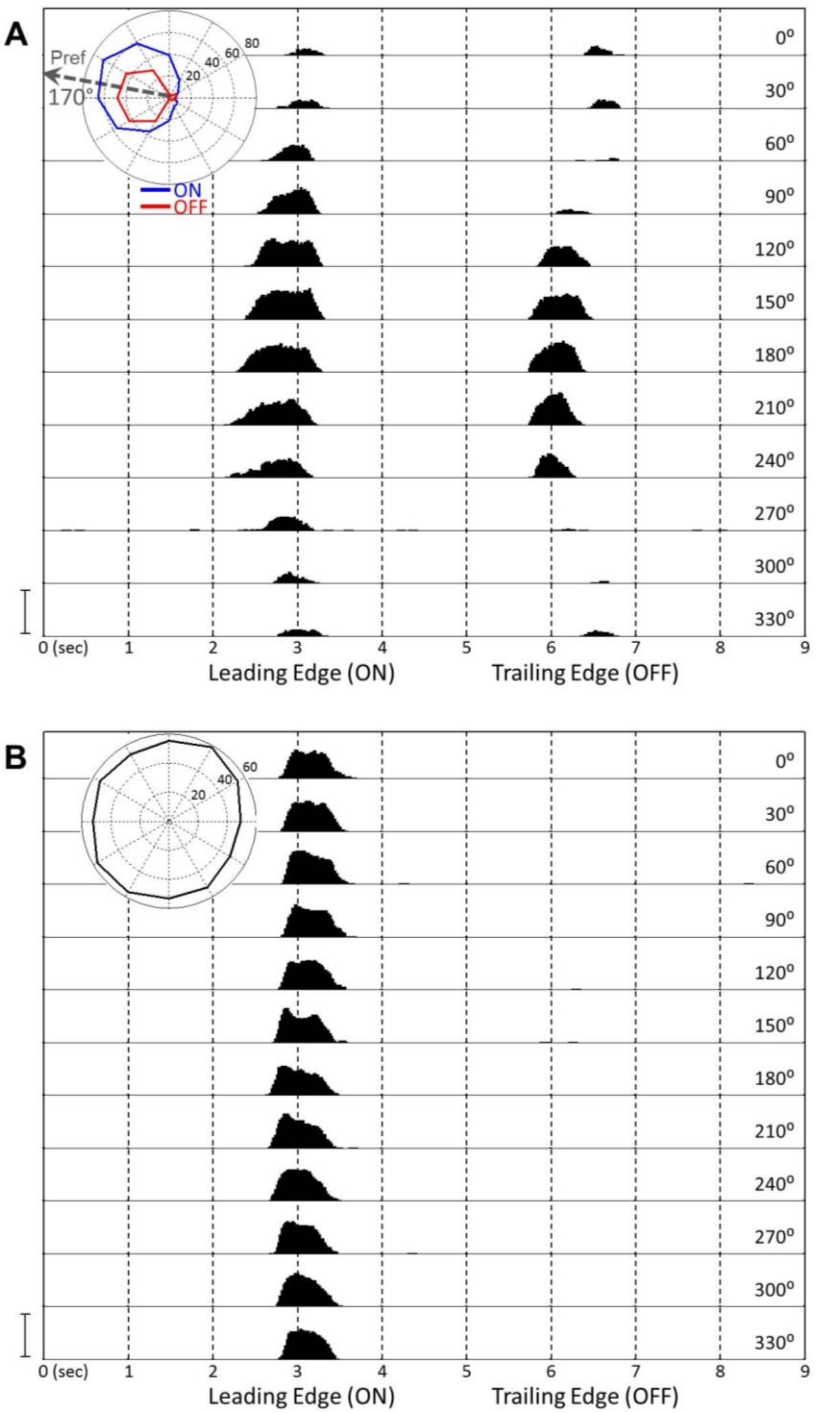

Fig. S1. Peristimulus time histogram (PSTH) in response to moving bars. (***A***) Responses of a typical ON-OFF DS cell in response to a white bar (300 × 1800 µm) moving at 600 µm/sec; responses to 12 different directions of motion were measured. (Inset) Total spike count as a function of movement direction (polar plot); ON and OFF responses plotted separately. Dashed arrow is the average of the vector sums for the ON and OFF plots. This is the same cell used in Fig. 1. (***B***) Response of a typical non-DS ON cell to moving bars. (Inset) Polar plot of ON responses. Scale bar, 150 Hz, applies to both panels.

A

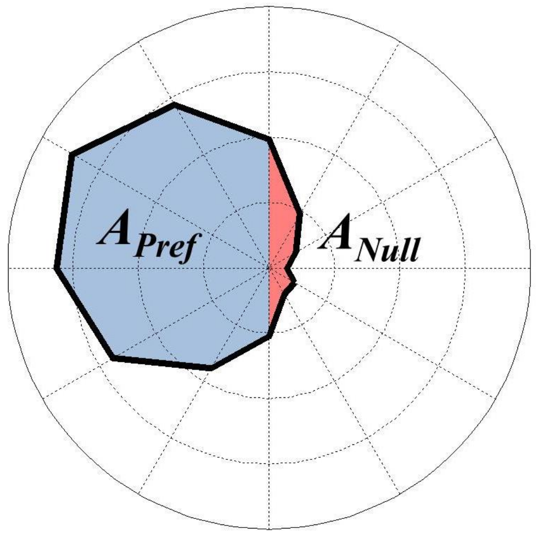

$$\text{Direction Selective Index (DSI)} = 1 - \frac{A_{Null}}{A_{Pref}} \ (= 0.924)$$

B

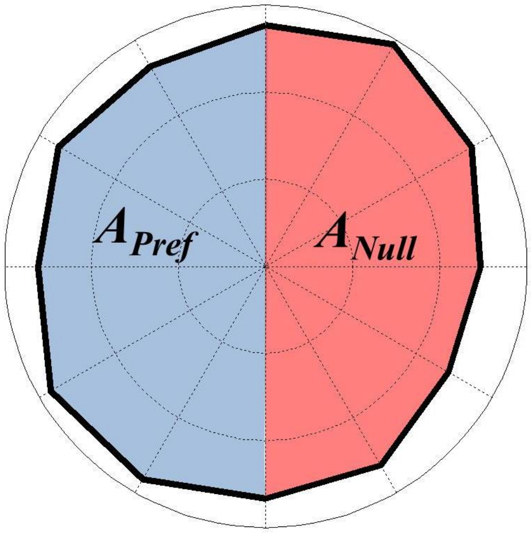

$$\text{Direction Selective Index (DSI)} = 1 - \frac{A_{Null}}{A_{Pref}} \ (= 0.084)$$

Fig. S2. Computation of the direction selective index (DSI). *DSI* was defined as $1 - A_{Null}/A_{Pref}$, where A_{Null} is null-side area and A_{Pref} is preferred-side area. (***A***) Sample computation for the ON responses of the DS cell used in Fig. 1. (***B***) Sample computation for the non-DS ON cell shown in Fig. S1*B*.

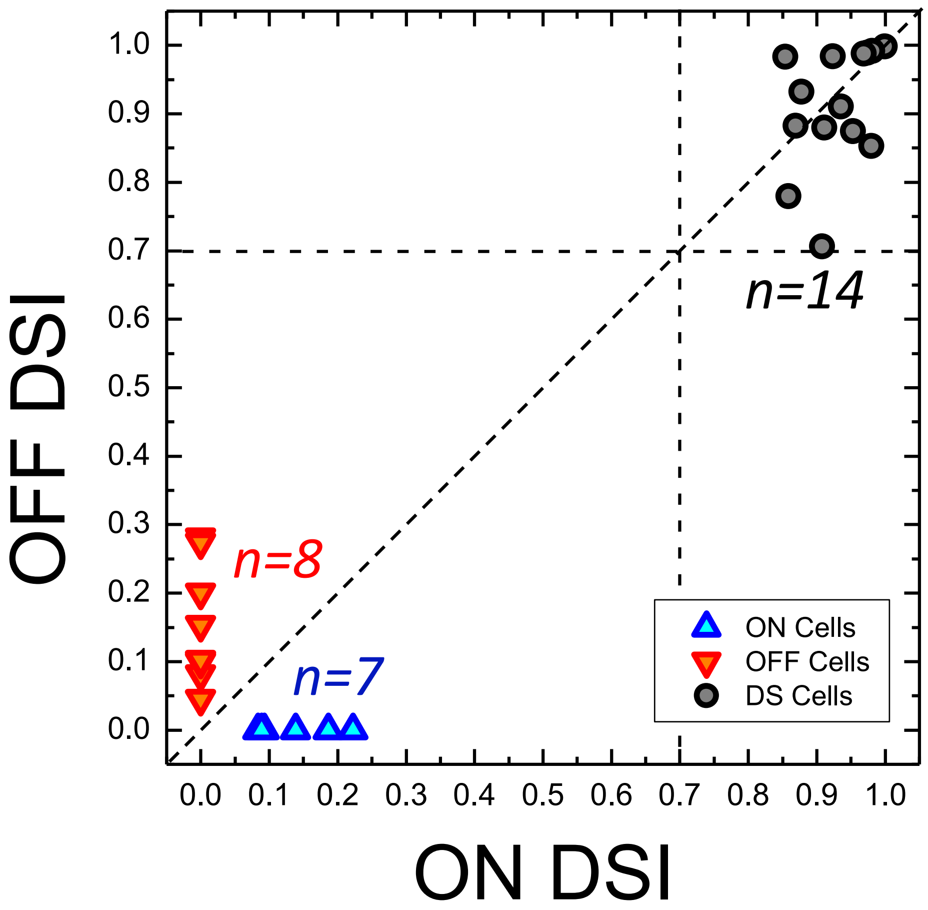

Fig. S3. Scatter-plot of direction selectivity indices (DSIs) of all cells used in this study. ON DSI and OFF DSI indicate DSIs associated with the leading (ON) and trailing (OFF) edges of the bright moving bars. Vertical and horizontal dashed lines indicate DSIs of 0.7 for ON and OFF responses, respectively. Diagonal dashed line is unity slope.

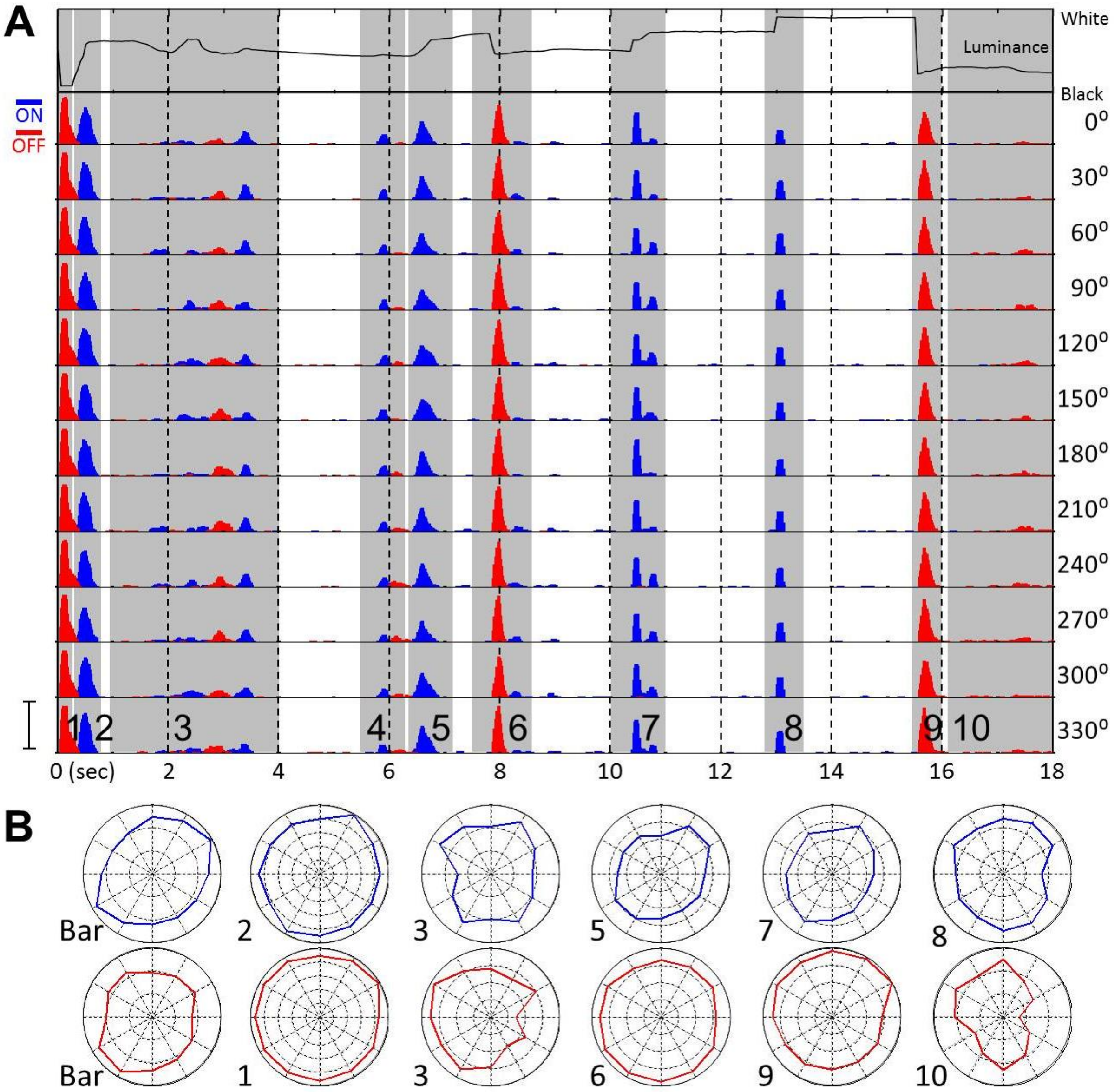

Fig. S4. Responses of non-DS ON and OFF cells to natural movies are symmetric. (***A***) Peristimulus time histogram (PSTH) of natural movie responses from one ON cell (blue) and one OFF cell (red); responses from the two cells are overlaid. Top row is the luminance profile corresponding to the central 300 μm of the movie. Scale bar, 300 Hz, applies to all rows. (***B***) Polar plots calculated for each scene from one of the two cells. Leftmost polar plots derived from the response to moving bars for the two cells. Scene numbers are shown at bottom left of each polar plot.

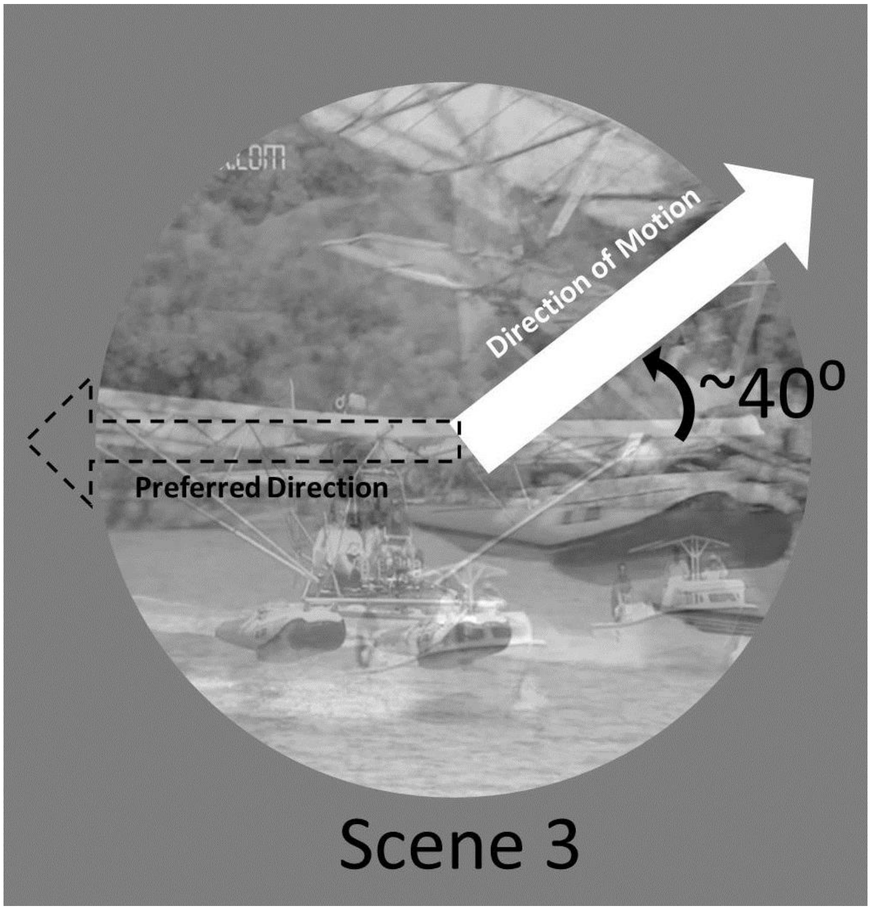

Fig. S5. Overlay of the two movie frames that correspond to the start and end of Scene 3. Solid arrow indicates approximate principal direction of the plane's motion, and dashed arrow indicates the cell's preferred direction (calculated from moving bar responses). The movie frames are from '*CORAL REEF ADVENTURE*' © MacGillivray Freeman Films. Used by permission. All rights reserved. This figure is not covered by the CC BY license.

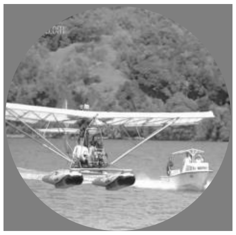

Whole movie ($2{,}000 \mu m$)

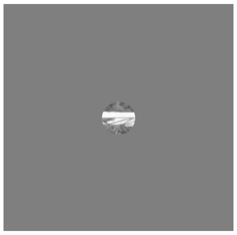

Masked movie ($300 \mu m$)

Fig. S6. Movie was shown either in its entirety (left) or with all but the central 300 μm of the movie masked (right) for luminance response suppression test shown in Fig. 2. The movie frame is from '*CORAL REEF ADVENTURE*' © MacGillivray Freeman Films. Used by permission. All rights reserved. This figure is not covered by the CC BY license.

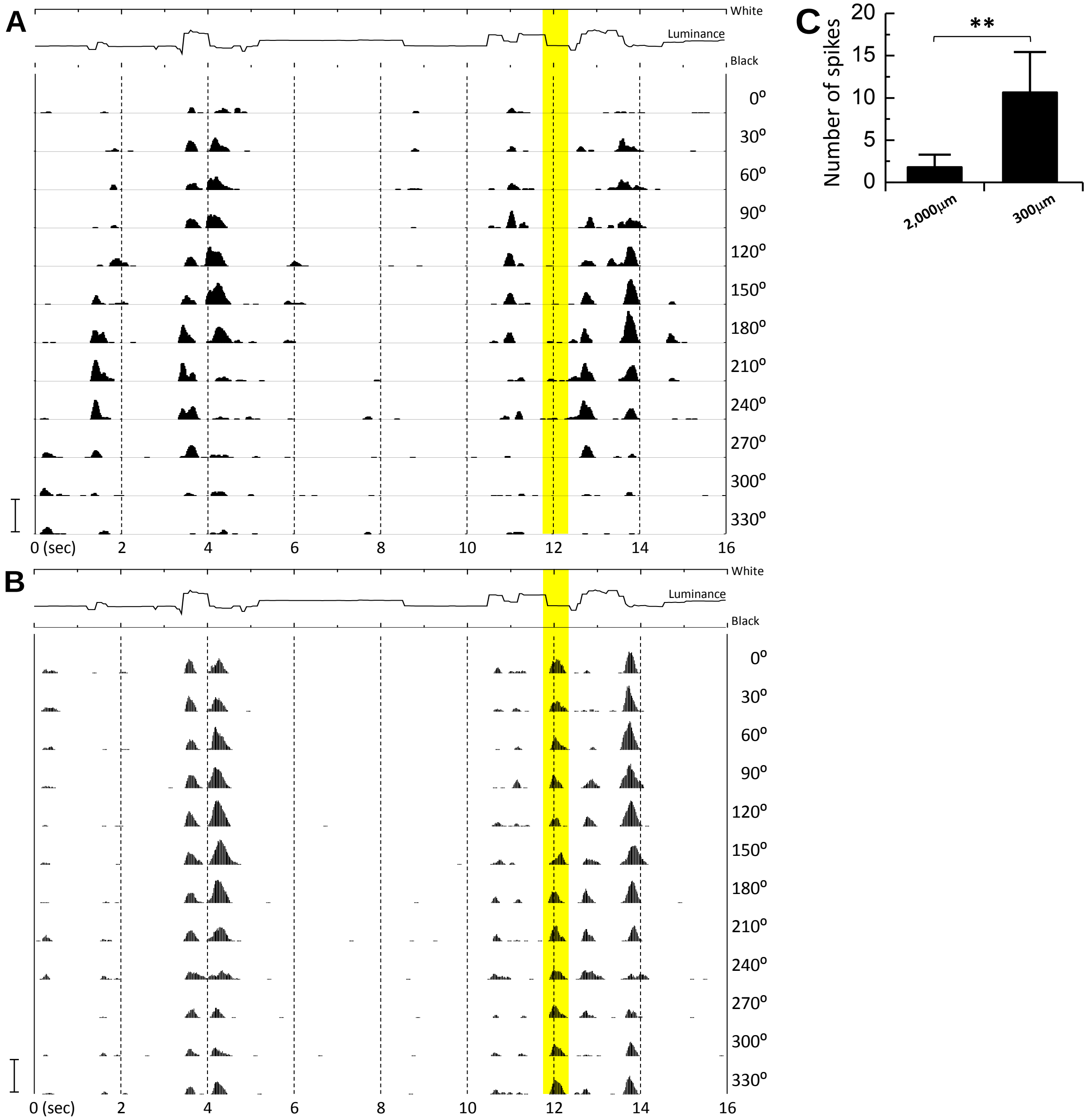

Fig. S7. Luminance responses are suppressed in DS cells in response to another natural movie. (***A***) Peristimulus time histogram (PSTH) from a DS cell in response to a natural movie shown in its entirety; each row corresponds to a different orientation of the movie (right). Scale bar at bottom left (150 Hz) applies to all rows. (***B***) Same as (*A*) but for the movie masked except the central 300 μm. (***C***) Average numbers of spikes elicited during the scene highlighted (yellow) in (*A*) and (*B*) for full and masked movies. In DS cells (n = 6), responses were reduced by 84% (masked vs. full). Paired one tailed student's t-test was applied to verify the significance of statistical comparisons; **P<0.01. Error bars, SD. Note that other large luminance decreases that were accompanied by motion (i.e. the scene that occurred ~ 4 sec) remain directional during both unmasked and masked presentations.

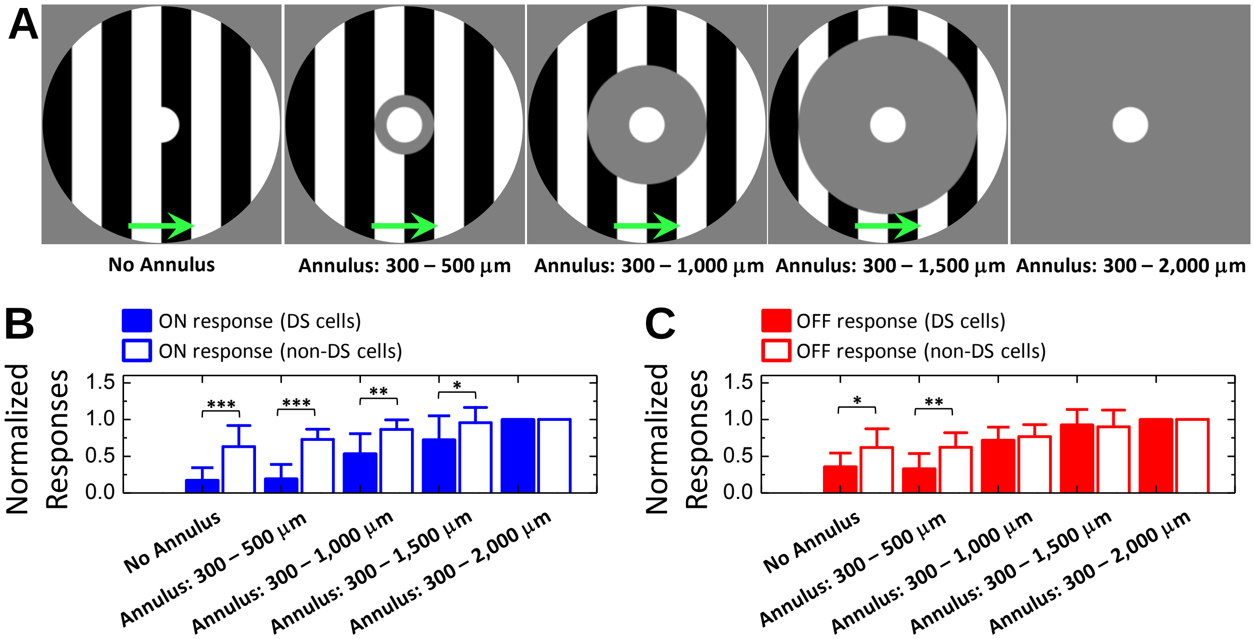

Fig. S8. Luminance responses are suppressed in direction selective ganglion cells more than in non-direction selective cells. (***A***) Spot flash of 300 µm in diameter was shown in presence of moving grating (2 cycles/mm and 2 cycles/sec) at surround. Gray annuli were used to mask increasing portions of the surround. Moving direction of gratings was indicated with a green arrow and rotated in 12 directions. Inner diameter of all annuli remained fixed at 300 µm while outer diameter ranged from 500 to 2,000 µm. (***B***) Average ON responses to white spot flashes as a function of annulus size in DS cells (n =12) and non-DS ON cells (n = 10). All responses were averaged across responses to 12 rotations and then were normalized to the number of spikes elicited in response to spot flashes without gratings (rightmost panel in *A*). (***C***) Same as (*B*) but for average OFF responses to black spot flashes in DS cells (n = 10) and non-DS OFF cells (n = 8). Paired one tailed student's t-test was applied to verify the significance of statistical comparisons; * $P<0.05$, ** $P<0.01$ and *** $P<0.001$. Error bars, SD (*B*, *C*).

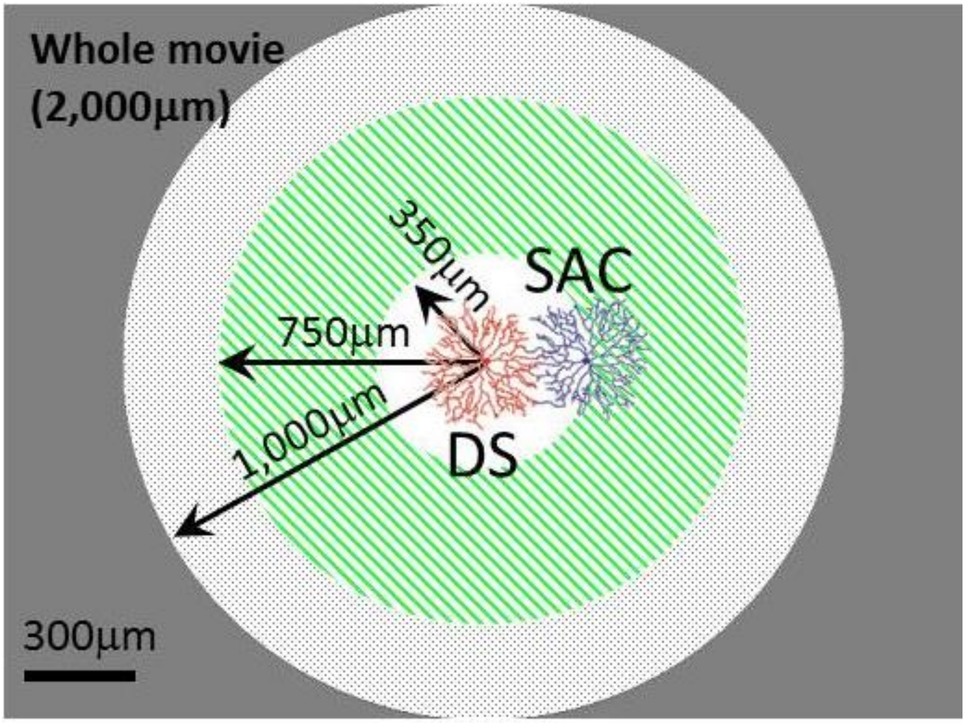

Fig. S9. Schematic comparison of movie size to dendritic field sizes. Dendritic field sizes of DS cells (red) were ~300 µm (diameter) while those of starburst amacrine cells (SACs, blue) were ~400 µm (diameter); thus the extent over which SACs deliver input to a DS cell is ~350 µm (radius, inner white circle). Suppressive effects were observed for increases in the annulus up to 750 µm (radius, green hatched region). The movie extended for 1,000 µm (radius, gray dotted region).

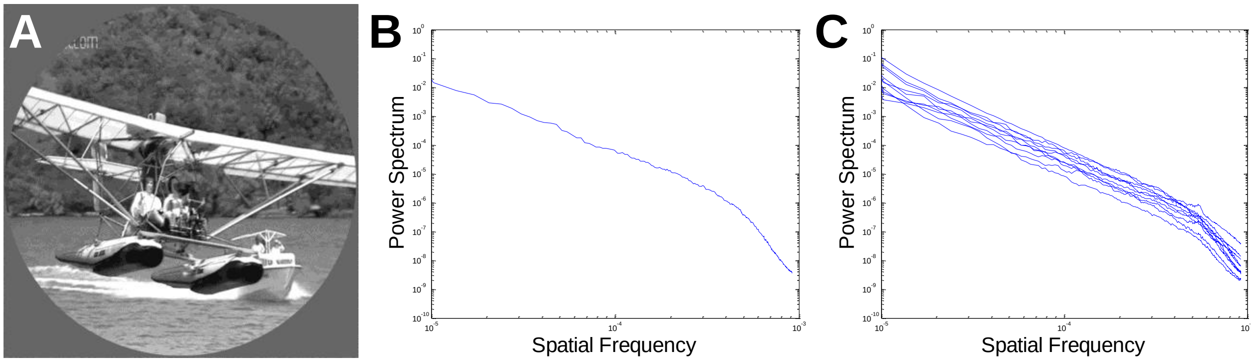

Fig. S10. **Natural movie shows characteristic power spectrum profile**. (***A***) A movie frame is shown from Scene 3. The movie frame is from ‘*CORAL REEF ADVENTURE*’ © MacGillivray Freeman Films. Used by permission. All rights reserved. This figure is not covered by the CC BY license. (***B***) Power spectrum of the image shown in (*A*) as a function of spatial frequency. (***C***) Same as (*B*) but for frames from other Scenes.

Artificial image (Laboratory stimulus)	Power spectrum profile
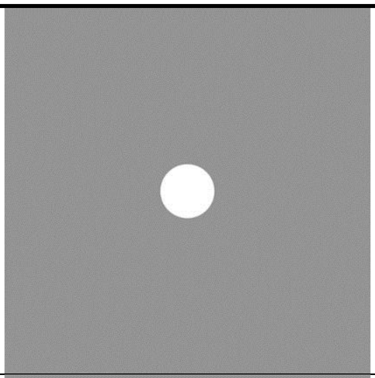	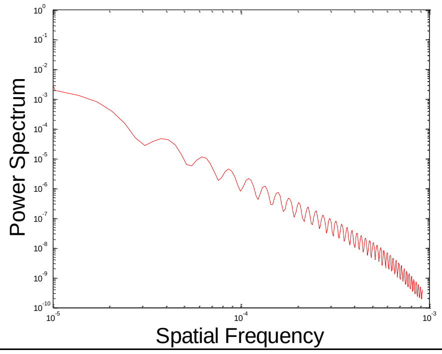
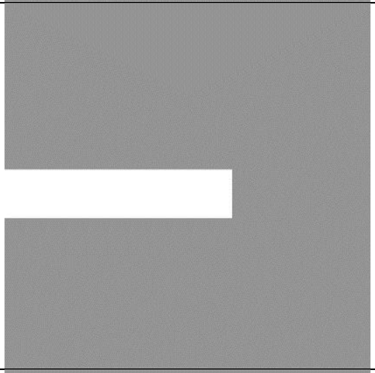	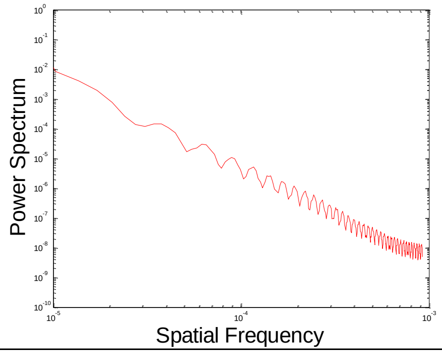
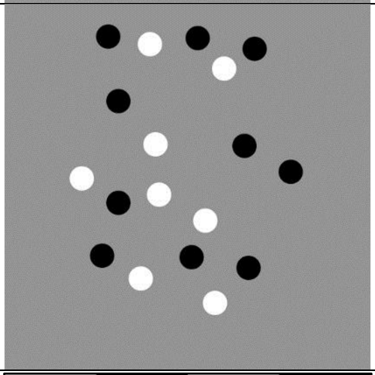	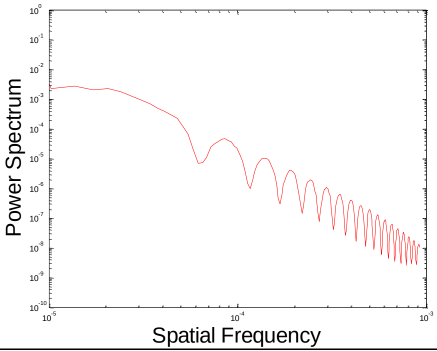
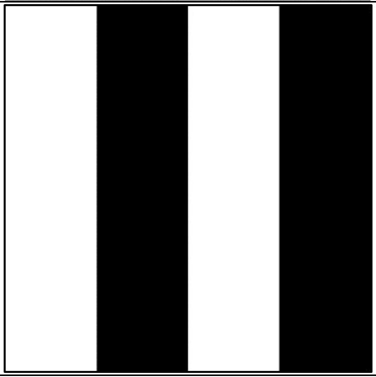	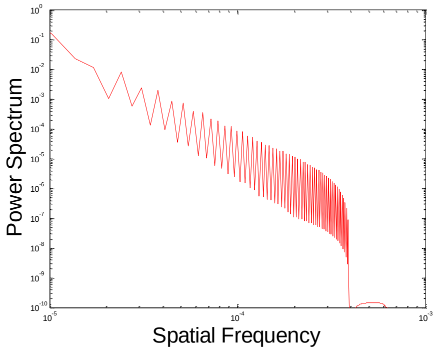

Fig. S11. **Power spectrum profiles of artificial images are different from those of natural images**. Left column shows some commonly used laboratory stimuli and right column shows their power spectra.

Movie S1. The naturalistic movie referred to in the text. The official movie trailer of '*CORAL REEF ADVENTURE*' © MacGillivray Freeman Films. Used by permission. All rights reserved. This video is not covered by the CC BY license.

Movie S2. The naturalistic movie referred to in the Supplementary Information. The animal documentary film of '*World's Deadliest: Stoat Hypnotizes Rabbit*' produced by National Geographic Society, licensed the footage directly from the BBC Motion Gallery. Used by permission from BBC Motion Gallery / Getty Images. All rights reserved. This video is not covered by the CC BY license.
